# New Era of Mapping and Understanding Common Fragile Sites: An Updated Review on Origin of Chromosome Fragility

**DOI:** 10.3389/fgene.2022.906957

**Published:** 2022-05-20

**Authors:** Fang Ji, Xinli Zhu, Hongwei Liao, Liujian Ouyang, Yingfei Huang, Madiha Zahra Syeda, Songmin Ying

**Affiliations:** ^1^ International Institutes of Medicine, The Fourth Affiliated Hospital of Zhejiang University School of Medicine, Yiwu, China; ^2^ Department of Pharmacology and Department of Respiratory and Critical Care Medicine of the Second Affiliated Hospital, Key Laboratory of Respiratory Disease of Zhejiang Province, Zhejiang University School of Medicine, Hangzhou, China; ^3^ Department of Respiratory and Critical Care Medicine of the Second Affiliated Hospital, Key Laboratory of Respiratory Disease of Zhejiang Province, Zhejiang University School of Medicine, Hangzhou, China

**Keywords:** common fragile sites, replication stress, chromosome fragility, high-resolution mapping, cancer

## Abstract

Common fragile sites (CFSs) are specific genomic loci prone to forming gaps or breakages upon replication perturbation, which correlate well with chromosomal rearrangement and copy number variation. CFSs have been actively studied due to their important pathophysiological relevance in different diseases such as cancer and neurological disorders. The genetic locations and sequences of CFSs are crucial to understanding the origin of such unstable sites, which require reliable mapping and characterizing approaches. In this review, we will inspect the evolving techniques for CFSs mapping, especially genome-wide mapping and sequencing of CFSs based on current knowledge of CFSs. We will also revisit the well-established hypotheses on the origin of CFSs fragility, incorporating novel findings from the comprehensive analysis of finely mapped CFSs regarding their locations, sequences, and replication/transcription, etc. This review will present the most up-to-date picture of CFSs and, potentially, a new framework for future research of CFSs.

## 1 Introduction

**FIGURE 1:**
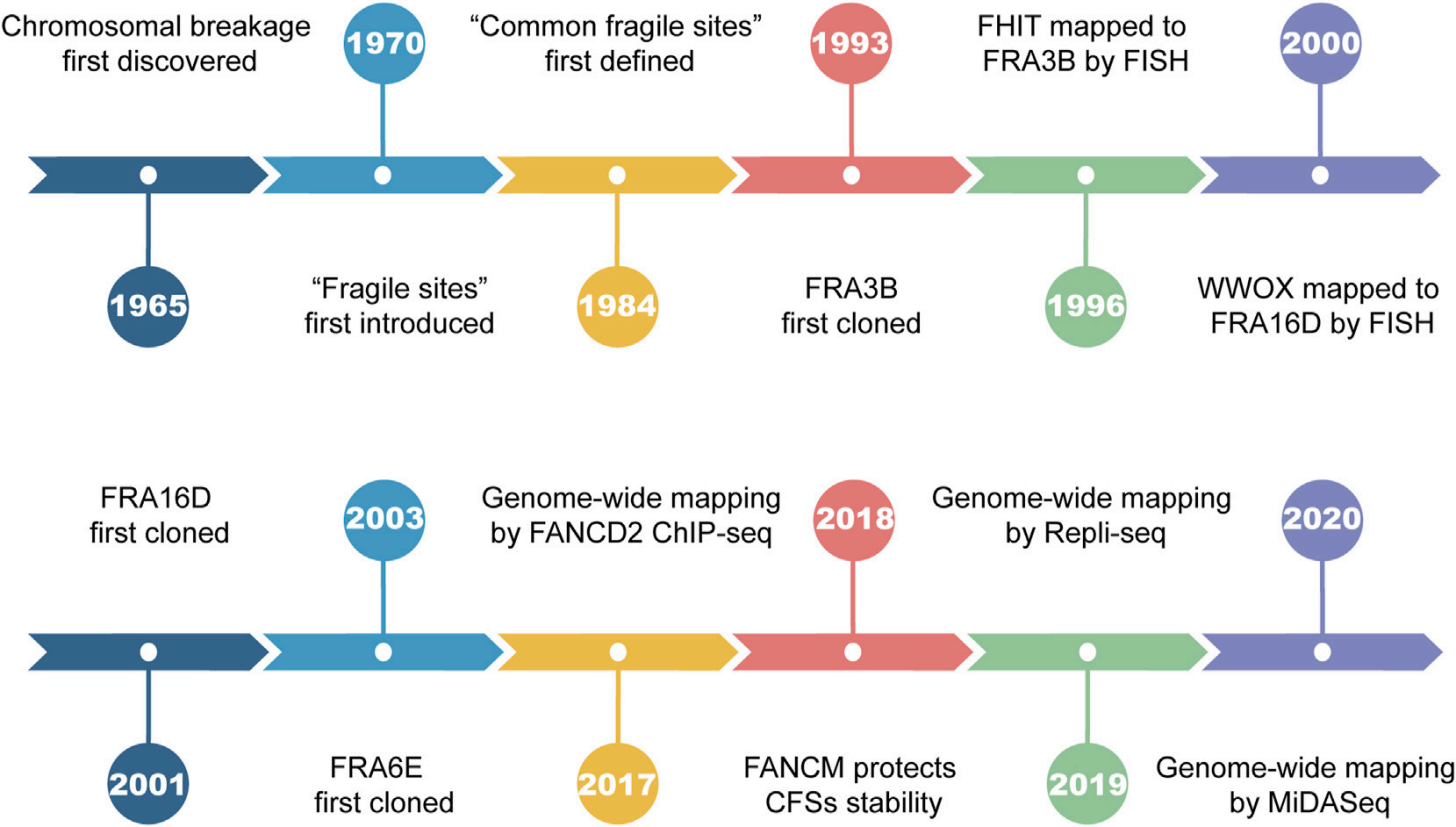
Landmark events in the history of common fragile sites.

Chromosomal breakage was first discovered by Dekaban *et al.* in 1965 during karyotype analysis of blood lymphocytes (Dekaban, 1965). The term “fragile sites” was first introduced to describe the recurrent breakage on the long arm of chromosome 16, which segregates in Mendelian fashion (Magenis et al., 1970). In the years that followed, the formation of fragile sites remained an enigma since it was not seen by all of the laboratories. Until 1977, the culture medium was suggested to play an important role in causing chromosomal breakage (Sutherland, 1977), explaining the disparity among laboratories. By 1983, 17 heritable fragile sites had been identified, 14 of which were induced by thymidylate stress such as folate deficiency, inhibition of thymidylate synthetase, or dihydrofolate reductase (Giraud et al., 1976; Sutherland, 1979; Glover, 1981; Sutherland, 1983). In 1984, Glover *et al.* revealed that site-specific breakage formed in all individuals upon exposure to a low dose of aphidicolin (APH), a classic DNA polymerase inhibitor that suppresses DNA synthesis (Glover et al., 1984). Their breakage frequency was found to be positively correlated with APH concentration and further increased under folic acid deprivation. The term “common fragile sites” (CFSs) was coined for the first time to define such chromosomal loci susceptible to APH treatment (Figure 1). Up to now, several chemical agents are known to be capable of inducing CFSs breakages (namely CFSs expression), such as APH, fluorodeoxyuridine (FUdR), 5-azacytidine (Aza), bromodeoxyuridine (BrdU), and distimycin A (Sutherland, 1982; Jacky and Sutherland, 1983; Reidy et al., 1983; Glover et al., 1984; Hecht et al., 1988; Rao et al., 1988).

In addition to CFSs, there are another two classes of chromosomal fragile sites, known as rare fragile sites (RFSs) and early replicating fragile sites (ERFSs). RFSs can only be detected in a limited number of individuals (less than 5%). Unlike CFSs, which are part of normal chromosomal structure and span a genomic region of several kilobases with high breakage probability, RFSs are linked to specific sequence repeats, such as CGG-repeat, and the breakage frequency is dependent upon the expansion of repeats (Zlotorynski et al., 2003; Schwartz et al., 2006). ERFSs locate in early-replicated chromosomal regions with high GC content and contain high densities of replication origins (Barlow et al., 2013; Mortusewicz et al., 2013; Voutsinos et al., 2018), all of these features are distinct from CFSs.

Early studies utilized the positional cloning approach to directly clone and characterize CFSs, which is based on large-insert clones (such as yeast artificial chromosomes). For example, FRA3B, the most frequently expressed CFS in human lymphocytes, was first cloned in 1993 (Boldog et al., 1993; Arlt et al., 2003) and is located within the tumor suppressor gene *FHIT* (Smeets et al., 1986; Negrini et al., 1996). FRA16D, the second most active CFS located within the *WWOX* gene, was first cloned by Paige *et al.* in 2001 (Sutherland and Richards, 1995; Krummel et al., 2000; Paige et al., 2001). For decades, a great deal of effort has been devoted to identifying CFSs, associated genes, and the underlying mechanism of CFSs instability. In this review, we will focus on the methodologies used to map CFSs and new insights discovered.

## 2 Biological Relevance of CFSs

It has been well documented that CFSs play an important role in chromosomal structural rearrangements and copy number variation (CNV) during tumor progression. The relationship between CFSs and cancer can date back to 1975 when Nemat *et al.* discovered that the fragility and breakage-prone nature of CFSs may explain the genetic transmission of retinoblastoma (Hashem and Khalifa, 1975). Many typical CFSs related genes, such as *WWOX* and *FHIT*, have been suggested as tumor suppressor genes, whose deletion and mutation may promote tumorigenesis and be linked to poor clinical outcomes (Huebner et al., 1997; Lee et al., 2001; Del Mare et al., 2016; Chang et al., 2018; Chen Y.-A. et al., 2019). *WWOX* has been found to have allele translocation and heterozygous loss in a variety of malignancies, including lung, kidney, breast, and prostate cancer (Paige et al., 2000). For the first time, Durkin *et al.* provided direct evidence that replication stress can induce submicroscopic deletions within FRA3B, which are similar to those found in both normal and tumour cells (Durkin et al., 2008). Besides, correlation analysis revealed that CNV hotspots correspond to CFSs in a given cell line exemplified by the human 090 fibroblast cell line, which can not be explained by a coincidence (Wilson et al., 2015).

In addition to their important role in cancer, CFSs associated genes are also involved in neurological development. *PARKIN* was the first classic fragile gene reported to have neuroprotective functions, whose deficiency leads to autosomal recessive juvenile parkinsonism (Lorenzetti et al., 2004). The constitutional deletions within *AUTS2*, *IMMP2L,* and *NRXN1* have been associated with autism spectrum disorder, intellectual disability, and psychiatric disorders (Gregor et al., 2011; Swaminathan et al., 2012; Nagamani et al., 2013). Likewise, *WWOX* was also implicated in neuronal differentiation and development, whose genetic deficiency causes epilepsy, intellectual disability and degenerative neuropathies (Wang et al., 2012; Sze et al., 2020). Most recently, the *WWOX* gene has been listed as one of the risk factors for Alzheimer’s disease by a genome-wide association meta-analysis (Kunkle et al., 2019). Many other large CFS genes also appear to function in neurological development, such as *GRID2*, *CNTNAP2,* and *DMD*.

Besides, early cytogenetic studies suggested that CFS regions correlated with viral integration sites (Cannizzaro et al., 1988; Popescu and DiPaolo, 1989; Popescu et al., 1990; Matovina et al., 2009). Considering that integration of DNA viruses into the human genome is a prerequisite for malignant transformation, CFSs may facilitate tumorigenesis by providing target sites for viral integration. Using fluorescence *in situ* hybridization (FISH) analysis, Wilke *et al.* provided direct evidence that FRA3B coincides with spontaneous human papillomavirus 16 (HPV16) integration site in a primary cervical carcinoma (PCC) for the first time (Wilke et al., 1996). Furthermore, genome-wide profiling of viral integration on HPV-positive cell lines revealed an HPV integration landscape and proposed a model in which microhomology-mediated DNA repair pathways may drive the incorporation of viral DNA into the human genome (Hu et al., 2015). Interestingly, mate-pair next-generation sequencing (MP-seq) revealed that CFSs and associated large genes are preferential sites for HPV integration and chromosomal rearrangements in oropharyngeal squamous cell carcinoma (OPSCC) (Gao et al., 2017). It was reported that hepatitis B virus (HBV) integration occurred within or near fragile sites, such as FRA16E and FRA7J (Bonilla Guerrero and Roberts, 2005; Feitelson and Lee, 2007). However, a meta-analysis showed that HPV and HBV integration were not correlated with fragile sites (Doolittle-Hall et al., 2015).

## 3 Genetic Mapping of CFSs: Gaining Insights Into the Nature of CFSs Fragility

**FIGURE 2:**
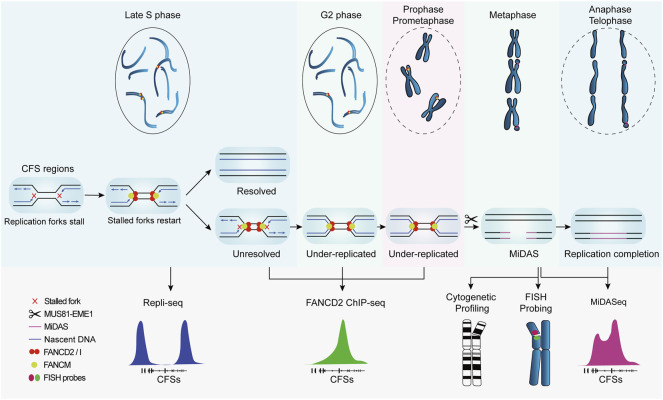
Replication profile of common fragile sites and corresponding methods for genetic mapping. CFSs tend to replicate at the late S phase. Under replicative stress, replication forks at CFSs slow down and stall. FANCM localizes to stalled forks and promotes fork reversal and restart, preventing DSB formation and CFSs instability. If the stalled forks are not resolved, CFSs remain under-replicated until mitosis. Besides, FANCD2/I complex is also recruited to CFS regions and persists into mitosis to protect the under-replicated intermediates. MUS81-EME1 endonuclease complex actively cleaves under-replicated intermediates and leads to DSB formation, which is cytogenetically visible at metaphase chromosomes. Thereafter, mitotic DNA synthesis (MiDAS) is carried out to finish the replication and thus facilitates chromosomal segregation and genome stability.

The advancement of knowledge on CFSs largely relies on correlating their genetic characteristics to their breakage and ensuing consequences. In this regard, genetic mapping that provides the coordinates and sequences of CFSs is crucial to the study of CFSs. In early studies, the cytogenetic assay was used to coarsely localize CFSs in individual chromosomes at the resolution of chromosome bands spanning several megabases, e.g., FRA3B in 3p14.2, FRA16D in 16q23 (Mrasek et al., 2010). Though feasible to perform and economically friendly, it provides no detailed genetic information such as associated genes and sequences that are crucial to understanding the origin of CFSs fragility. Therefore, fluorescence *in situ* hybridization (FISH) was introduced to characterize CFSs at the molecular level, mapping CFSs to specific genes, e.g., FRA3B to *FHIT* and FRA16D to *WWOX*. Recently, by virtue of the progress in understanding of CFSs formation and revelation of CFS-specific biological events, high-throughput DNA sequencing based on surrogate protein markers (Okamoto et al., 2018; Pentzold et al., 2018) or temporally restricted DNA synthesis (Ji et al., 2020; Zhao et al., 2020), has led to a more efficient approach to mapping CFSs at nucleotide resolution, which provides many new insights into the origin of CFSs fragility, although it should be noticed that CFSs mapped in cell populations, which are based on assumptions that stalled replication forks and breakages in DNA are bound to manifest as visible gaps on metaphase chromosomes, are semantically controversial and should not be considered equating to the incipiently defined ones until experimentally validated to occupy cytogenetically visible breaks in single cells. In the following sections, we will introduce the methodologies and principles of CFSs mapping by FISH and recently developed high-throughput sequencing (Figure 2), and provide a comparative analysis of their advantages and limitations to help readers to adopt the optimal method for their research of interest.

### 3.1 Fluorescence *in Situ* Hybridization: FISHing CFSs out

Fluorescence *in situ* hybridization (FISH) of CFS regions of megabases is made possible by the establishment of bacterial/yeast artificial chromosome libraries. These libraries based on the human genome project have provided great convenience for preparing FISH probes with ultra large sizes. The principle of FISH is the specific base pairing between the targeted chromosomal region and fluorescent complementary probes. In a typical process of CFS characterization by FISH, complementary probes with known genetic sequences are used to mark and visualize the position of CFSs expressed on a metaphase chromosome, imparting the genetic association and coordinates of CFSs. Thus far, plenty of researches have applied FISH to investigate CFSs instability (Magenis et al., 1970; Paradee et al., 1995; Boldog et al., 1997; Le Tallec et al., 2011; Mrasek et al., 2015). It helps to determine whether a specific large gene lies within a CFS locus according to the relative position, classified as “proximal (centromeric)”, “crossing (on)”, and “distal (telomeric)”, of breakage regions toward hybridization signal of probes corresponding to the gene of interest. Statistical analysis of an adequate number of chromosome spreads then reveals the approximate location of large genes relative to CFSs, which can be further refined by using multiple shorter probes. FISH has contributed immensely to our knowledge on CFSs. It, however, has some ingrained disadvantages. For example, the FISH protocol is rather time-consuming, labor-intensive, and involves high denaturing temperature that may cause artifacts in chromosomal morphologies. Moreover, commercially available FISH probes, mainly from artificial chromosome libraries, only cover part of highly active human and murine CFSs, making it even more technically challenging to use FISH to map less expressed CFSs and CFSs in other species, as customized FISH probes may need to be prepared by the researchers.

### 3.2 High Throughput Sequencing: Mapping CFSs Globally

Given the multitude of CFSs and their wide distribution across the genome, it is unrealistic to use FISH or other conventional molecular biology techniques like molecular cloning to characterize and analyze all CFSs in one comprehensive study. Instead, only a few notorious CFSs have been intensively investigated, leading to inconclusive hypotheses that may not necessarily reflect the ubiquitous properties of most, if not all, CFSs. Thus, a comprehensive sequence analysis of multiple CFSs on a genome-wide scale is rather tempting, which may facilitate the formulation of general theories about the origin of CFSs. It was, however, extremely challenging to implement such a strategy in the early years, due to the lack of high-throughput experimental tools and, more importantly, poorly defined markers that can be used to screen most CFSs out from the genomic background. In recent years, decades of CFSs knowledge accumulation and the advent of high-throughput yet low-cost sequencing technologies have finally converged to permit for genome-wide analysis of CFSs, ushering CFSs research into a new era of precise mapping, comprehensive analysis, and, perhaps, accurate prediction of chromosome fragility. In the following sections, we will introduce different high-throughput sequencing approaches, with descriptions of the biological rationales that will facilitate understanding and critical assessment of these methodologies.

#### 3.2.1 Repli-seq

Repli-seq is a well-established approach to monitoring DNA replication dynamics at the different timepoint of the S phase on the genome-wide scale. It has been extensively applied for detecting replication timing (RT) program variation across multiple cell lines during physiological and pathological conditions, such as differentiation (Hiratani et al., 2008; Hansen et al., 2010; Ryba et al., 2011; Marchal et al., 2018; Zhao et al., 2020). This method generally starts with nascent DNA labeling using bromodeoxyuridine (BrdU) or other thymidine analogs that can be incorporated into the genome when DNA replication occurs. Then cells in different stages of the S phase, e.g., early S phase, middle S phase, and late S phase, will be collected by fluorescence-activated cell sorting. The newly synthesized DNA labeled with thymidine analogs can be enriched by antibody-based immunoprecipitation or click chemistry-based separation. By sequencing the nascent DNA, a map of the actively replicating regions in different stages of the S phase can be obtained.

Since delayed replication upon replication stress is a typical feature of CFSs, the Repli-seq profile from the “late S phase” cells can largely mark the genetic locations of CFSs (Brison et al., 2019; Sarni et al., 2020). Using this method, Brison *et al.* generated a genome-wide atlas of CFSs in human lymphoblastoid JEFF cells for the first time, including 32 CFSs accounting for 82 percent of breakage sites cytogenetically identified as CFSs (Brison et al., 2019). Another group also mapped CFSs using Repli-seq in immortalized human fibroblasts (Sarni et al., 2020). They revealed that CFSs, even their core regions, are also replicated at early/mid S phases in the absence of replication stress. This implies that late replication timing is not the intrinsic property of CFSs, but an acquired one under replication stress.

Hopefully, Repli-seq will lead to more novel findings in the future. However, it should be noted that delayed replication is necessary but insufficient to induce CFSs instability. Therefore, combination with other methodologies such as FISH is required to validate whether a suspected fragile site revealed by Repli-seq is a bona fide CFS exhibiting breakage on metaphase chromosomes.

#### 3.2.2 FANCD2 ChIP-seq

Fanconi anemia complementation groups (FANC) proteins are well-known to carry out major functions in repairing DNA interstrand crosslinks (ICLs), whose deficiency causes the cellular hypersensitivity to ICL-inducing agents, such as mitomycin C (MMC) or diepoxybutane (DEB) (Moldovan and D'Andrea, 2009; Liu et al., 2010; Kottemann and Smogorzewska, 2013). The genetic defect of FANC proteins causes Fanconi anemia (FA), a rare genetic disease characterized by DNA damage accumulation, bone marrow failure, congenital abnormalities, and cancer predisposition (Ceccaldi et al., 2016). In the context of DNA damage repair, the FA core complex (comprised of FANCA, FANCB, FANCC, FANCE, FANCF, FANCG, and FANCM) is recruited to stalled replication fork and monoubiquitinates FANCD2/I complex. Subsequently, the monoubiquitinated FANCD2/I complex promotes interstrand cross-linking incision by coordinating with other nucleases named XPF, MUS81, and SLX1, generating a double-strand break (DSB) to be repaired by ensuing homologous recombination (Moldovan and D'Andrea, 2009). While FANCD2 has long been known to play a role in ICL repair and maintaining replication progression by e.g., stalled replication fork stabilization (Schlacher et al., 2011; Schlacher et al., 2012; Sato et al., 2016), its biological relevance to CFSs stability was not brought into focus until the observation that lymphoblasts from FA-deficient patients showed a higher incidence of CFSs expression (Howlett et al., 2005). It has been well documented that replication stress induces localization of FANCD2/I twin foci to under-replicated regions of CFSs as early as the G2 phase, which can persist into anaphase if the under-replicated intermediates of CFSs are not timely resolved (Howlett et al., 2005; Chan et al., 2009; Naim and Rosselli, 2009). FANCD2 has recently been found to serve as a trans-acting facilitator of CFS replication, which promotes the efficient firing of origins and timely removal of DNA: RNA duplex (namely R loop) to ensure replication completion at CFSs before the onset of mitosis (Garcia-Rubio et al., 2015; Madireddy et al., 2016; Okamoto et al., 2019).

FANCM, another component of the FA core complex, is involved in ICL repair (Xue et al., 2008), replication remodeling, and telomere protection (Pan et al., 2017; Domingues-Silva et al., 2019; Pan et al., 2019; Silva et al., 2019). In Alternative Lengthening of Telomeres (ALT) cells, FANCM localized to telomeres, monitors telomeric replicative stress and promotes cell proliferation by R loop removal, whose deficiency causes uncontrolled telomeric stress, pronounced ALT activities, and cell death. Recent studies have revealed that FANCM has previously unappreciated roles in regulating repair pathways at stalled replication fork (Panday et al., 2021) and protecting CFSs stability (Wang et al., 2018). Mechanically, FANCM localized to stalled replication fork and recruits FA core complex through its MM1 domain, further facilitating FANCD2 ubiquitination and error-free homologous recombination repair (namely “short tract” gene conversion (STGC)). Besides, the MM2 domain of FANCM is indispensable for BLM interaction and suppressing the formation of error-prone homologous recombination repair, such as “long tract” gene conversion (LTGC) and non-homologous tandem duplications (TDs). On the other hand, using a mitotic recombination assay, Wang *et al.* found that FANCM prevents the formation of double-strand break at CFSs and ensuing mitotic recombination (Wang et al., 2018). Upon replication stress and oncogene expression, FANCM is recruited to CFSs and suppresses breakage formation by its translocase activity rather than the FA core and FANCD2/I complex.

Because of the high specificity of FANCD2 binding to CFSs under replication stress, FANCD2 chromatin immunoprecipitation sequencing (FANCD2 ChIP-seq) has recently been performed to map CFSs in a genome-wide manner. The overall procedure of ChIP-seq can be divided into six parts: crosslinking, chromatin fragmentation, antibody incubation, DNA elution, library construction and sequencing. Among these, antibody incubation is the key step which determines the specificity of enriched DNA. Therefore, the availability of FANCD2 antibodies with high affinity and specificity is of great importance. Pentzold *et al.* used FANCD2 ChIP-seq to mapped CFSs in an avian cell line and analysed several genetic characteristics to find out the decisive factor contributing to CFSs fragility (Pentzold et al., 2018). Their data supported that CFSs strongly associated with large genes with long transcripts and active transcription is required to induce fragility. Notably, a subset of putative early replicating fragile sites (ERFSs), another type of fragile sites characterized by early replicating time, high expression level and high gene density were also mapped (Barlow et al., 2013; Pentzold et al., 2018). Likewise, another study mapped CFSs by FANCD2 ChIP-seq and suggested that FANCD2 accumulated at the middle region of CFSs-related genes, protecting CFSs by facilitating the clearance of R-loops (Okamoto et al., 2018).

Compared with Repli-seq, FANCD2 ChIP-seq does not require DNA labeling or cell sorting, which greatly simplifies the experimental procedure. However, there are several inevitable limitations. As mentioned earlier, a ChIP-grade antibody with a high level of specificity and sensitivity is critical for successful enrichment of the targeted DNA, which directly determines the readout of sequencing. But such antibodies may not always be commercially available, especially for rare species. Therefore, researchers may need to develop high-performance antibodies or construct tagged FANCD2-expressing cell lines, both of which require a substantial commitment of time, labor and investment. Moreover, FANCD2 may preferentially function in a subset of CFSs protection, and is known to participate in repairing some types of DNA damage (Liu et al., 2010; Alcon et al., 2020), leading to bias and noise in CFSs mapping. As a result, a combination of FANCD2 ChIP-seq and FISH is necessary which helps to determine the precise coordinates of truly broken CFSs.

#### 3.2.3 MiDASeq

Since the discovery of CFSs, a great of effort has been devoted to dissect the molecular mechanism of chromosomal instability at CFSs. In early years, the cytogenetic breakage at CFS loci was considered to be an undesirable outcome of chromatid rupture during chromosome condensation in mitosis. However, Ying *et al.* discovered that it’s an active cleavage process mediated by the MUS81-EME1 complex, which serves to protect genomic stability and thus facilitates cell survival (Ying et al., 2013). They proposed such a model in which MUS81-EME1 complex localized to CFSs in prophase/prometaphase and cleaves the intertwined sister chromatid, resulting in CFSs breakage and faithful segregation of sister chromatid. A failure of cleavage induces the formation of ultra-fine anaphase bridges (UFBs) or bulk bridges at CFSs (Chan et al., 2009; Ying et al., 2013), relying on BLM and PICH to resolve (Baumann et al., 2007; Chan et al., 2007; Nielsen et al., 2015; Nielsen and Hickson, 2016). Unresolved UFBs and bulky bridges can trigger chromosome mis-segregation and genomic instability (Minocherhomji and Hickson, 2014). An even more striking finding revealed that CFSs undergo nascent DNA synthesis during mitosis after breakage (Minocherhomji et al., 2015). Mitotic DNA synthesis (termed MiDAS) appears to be the last resort to complete the replication of CFSs before chromosomal segregation, which helps to maintain genomic integrity. Furthermore, several studies suggested that MiDAS is carried out by break-induced replication (BIR), a homologous recombination (HR)-based repair pathway for one-ended DSBs (Llorente et al., 2008; Costantino et al., 2014; Kramara et al., 2018). To summarize above findings, HR protein RAD52 promotes the recruitment of MUS81 to CFSs and timely formation of MUS81-EME1 endonuclease complex during mitosis (Bhowmick et al., 2016; Sotiriou et al., 2016). After that, RECQ5, a member of the RecQ family of DNA helicases, dismantles the RAD51-ssDNA filaments at CFSs, allowing MUS81-EME1 to initiate resection (Di Marco et al., 2017) and promoting POLD3-dependent MiDAS (Minocherhomji et al., 2015). Recently, the regulator of telomere elongation helicase 1 (RTEL1) has been identified as a novel player in preserving the integrity of CFSs, with the ability to reduce R loop formation and assist MiDAS by recruiting RAD52 and POLD3 (Wu et al., 2020).

Based on the above findings, we and another group have developed a novel method named MiDASeq that allows mapping of CFSs with a nucleotide resolution in a genome-wide manner (Ji et al., 2020; Macheret et al., 2020). It is achieved through direct sequencing of the genomic regions that undergo mitotic DNA synthesis. MiDASeq begins with inducing MiDAS occurrence at CFSs and labeling nascent DNA with EdU, a thymidine analog which can be incorporated into the genome during DNA replication. Secondly, mitotic cells are collected manually by shake-off and subjected to Click-IT reaction that conjugated a biotin molecule to EdU. Third, genomic DNA was isolated and fragmented by sonication. Then, biotinylated DNA was captured by streptavidin beads. Finally, DNA library was constructed and subjected to next generation sequencing (NGS).

Compared with Repli-seq and FANCD2 ChIP-seq, which map “suspicious” CFSs, MiDASeq is closer to mapping “convicted” CFSs that are truly expressed, and therefore has higher specificity. Moreover, MiDASeq enriches nascent DNA fragments through click reaction and biotin-streptavidin binding, both of which have great specificity and sensitivity. And this antibody-free system can be easily used across different species. The main limitation is that MiDASeq requires a large number of mitotic cells to start with, which largely relies on the high efficiency of cell synchronization. Of note, MiDASeq has identified many chromosome regions with a certain extent of cell type specificity that are not listed in the current database of CFSs (Kumar et al., 2019), implying that these regions may also exhibit fragility. Whether these sites undergo breakage needs further validation with conventional methods, such that the true power of MiDASeq can be revealed.

## 4 Updated Insights Into the Fragility of CFSs

### 4.1 Secondary Structure-Forming Sequences

CFSs are referred to difficult-to-replicate regions, and the sequence characteristics have been extensively studied to explain the cause. Till now, the most common sequence feature of well-studied CFSs has been high AT content. For example, the *FHIT* gene at FRA3B harbors much more AT-dinucleotide repeats than the flanking nonfragile *PTPRG* gene (Matsuyama et al., 2003). Such a feature is also shared by FRA7E (Zlotorynski et al., 2003), FRA16B (Burrow et al., 2010), and FRA16D. Using a yeast artificial chromosome breakage assay, the Freudenreich group for the first time identified an AT-rich sequence element called Flex1 within FRA16D, promoting the formation of breakage in an AT length-dependent manner (Zhang and Freudenreich, 2007; Kaushal et al., 2019). Likewise, FRA16C also contains AT-rich subregions and replicates slower than the bulk genome due to the frequent pausing of replication forks in that region (Ozeri-Galai et al., 2011). In an attempt to go from correlation to causality, Sinai *et al.* directly investigated the consequence of high AT content. They integrated a 3.4 kb long AT-dinucleotides rich sequence derived from FRA16C into a stable ectopic site on chromosome X and found that it did have the ability to drive fragility, even though breakage frequency is lower than that of FRA16C (Irony-Tur Sinai et al., 2019). This result strongly suggests that AT-dinucleotides account, at least in part, for the high instability of CFSs. The putative mechanism is that AT-rich regions have higher flexibility and are prone to forming secondary DNA structures, such as hairpins and cruciform. These self-folded structures can retard the progression of replication machinery, resulting in the difficult-to-replicate nature of CFSs. In the presence of exogenous replication stress that stalls or slows DNA polymerases, long stretches of ssDNA at AT-rich sites can arise from the DNA polymerase-helicase uncoupling, creating even more complex secondary structures that may completely block replication (Kaushal and Freudenreich, 2019; Lyu et al., 2019). The causal relationship between high AT content and CFSs formation seems to fit into a tempting model where the fragility of a CFS is written in its genetic sequence. However, this hypothesis may only apply to a subset of CFSs that have been tested experimentally. Counter arguments claim that CFSs fragility is independent of the DNA sequence based on the following observations. First, the specific breakage sites of the same CFS vary among different cell types or even different cell lines derived from the same tissue (Le Tallec et al., 2013). Secondly, the sequence analysis of CFSs identified by FANCD2 ChIP-seq found no significant sequence characteristics (Pentzold et al., 2018). Thirdly, the genome-wide mapping of CFSs using Repli-seq failed to associate aphidicolin-induced under-replicated regions with specific DNA sequences (Brison et al., 2019). Most recently, we mapped CFSs by MiDASeq and found that CFSs had significantly higher AT content and more AT-dinucleotides repeats than random sites do. However, there was considerable heterogeneity among CFSs. Another parallel study using the same method showed that no specific sequences could serve as site-specific roadblocks to stall replication forks within CFSs (Macheret et al., 2020). These data suggest that secondary structure-forming sequences like AT repeats may only be an enhancer of CFSs fragility, acting downstream of some epistatic factors driving the formation of CFSs.

### 4.2 Replication Deficit

It is widely accepted that CFSs are hypersensitive to DNA replication stress that induces replication fork slowing and stalling. When exposed to replication stress, CFSs often fail to complete their replication prior to mitotic entry, and consequently display as visible gaps or breakages on metaphase chromosomes. The replication dynamics of CFSs, including replication timing and origins, have been extensively studied to explain the instability of CFSs upon replication stress. Late replication is prevalent among CFSs, leading to a low tolerance to any disruption of the replication machinery (Durkin and Glover, 2007). For example, FRA3B was found to complete replication in the late S phase in human lymphocytes (Le Beau et al., 1998). Other CFSs, such as FRA16D (Palakodeti et al., 2004), FRA7H (Hellman et al., 2000), FRA1H (Pelliccia et al., 2008), and FRA2G (Pelliccia et al., 2008), also display similar late or slow replication patterns (Maccaroni et al., 2020). Very recently, genome-wide replication profiling has directly shown that 47 of 59 CFSs in human lymphoblastoid JEFF cells are replicated in the middle to late S phase, and remain under-replicated before entering mitosis upon replication stress, demonstrating late replication as a general feature of common fragile sites (Brison et al., 2019). Consistently, we and Macheret *et al.* came to the same conclusion by analyzing the replication timing of much more CFSs mapped by MiDASeq (Ji et al., 2020; Macheret et al., 2020). Notably, late replication of CFSs is conserved across species, as shown that the majority of CFSs mapped by FANCD2 ChIP-seq in avian cells were also late replicating (Pentzold et al., 2018). However, late replication is not sufficient to define CFSs, as certain genetic regions replicating late in the S phase are rather stable. In addition to late replicating, CFSs tend to be lack of replication origins. Using a high-throughput microarray-based probing, Palakodeti *et al.* first revealed that there were only four replication origins in a 50 kb region of FRA3B (Palakodeti et al., 2010). Moreover, they demonstrated that replication origins of FRA3B may have a lower firing efficiency, further increasing the risk of replication failure. The abundance of origins in the same CFS region may vary among different cell lines. Letessier *et al.* combined DNA combing and FISH to reveal that there were no initiation events within the 700-kb-long core region of FRA3B in human B lymphocytes, whereas over 10 active origins distributed evenly within the same region in MRC-5 fibroblasts where FRA3B was much more stable. Considering that FRA3B is late replicated in both cell types, they proposed that a paucity of initiation events and late replication timing collectively contributed to the fragility of FRA3B (Letessier et al., 2011). Replication profiling of other CFSs such as 3q13.3 and 1p31.1 led to similar conclusions (Le Tallec et al., 2011). Recently, genome-wide replication origin mapping using ChIP-seq of origin recognition complex (ORC) or mini chromosome maintenance 7 (MCM7) revealed that scarcity of active origins was prevailing in CFSs (Miotto et al., 2016; Sugimoto et al., 2018).

**FIGURE 3:**
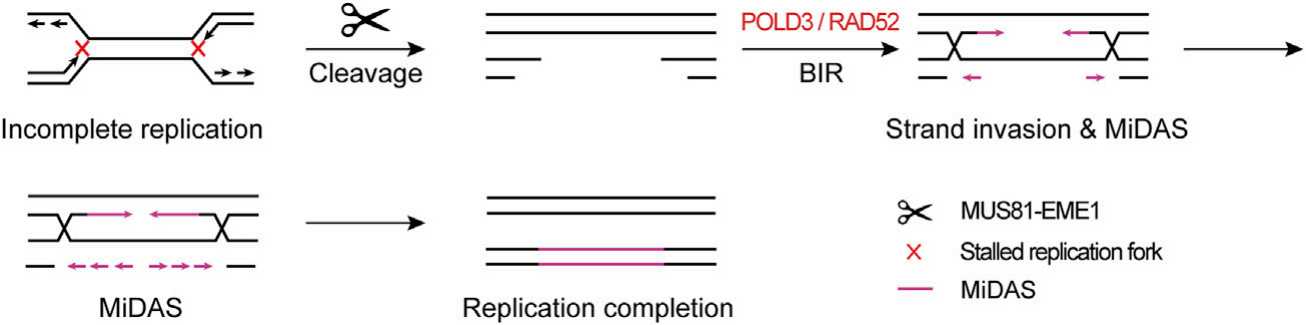
Model of “double fork failure” and “break-induced replication”. Under replication stress, both of the replication forks at CFSs stall, namely “double fork failure”, and CFSs remain under-replicated before mitosis. In prometaphase, both of the stalled forks are cleaved by MUS81-EME1 complex, giving rise to the DSB formation. Thereafter, two head-on forks restart replication through BIR, which is dependent on POLD3 and RAD52.

These findings point to a mechanistic model in which CFSs lie in large late-replicating regions that lack initiation events, relying on forks emanating from flanking regions to travel a long distance to complete the replication within a rather short time before mitosis, leading to the hypersensitivity of CFSs to, and instability under, replication stress. If both of the converging replication forks are stalled, namely “double fork failure”, the CFSs will remain under-replicated until mitosis. Afterwards, MiDAS serves to finish the replication by BIR. This model is well supported by the “twin peak” pattern of DNA signals in CFSs mapped by Repli-seq and MiDASeq in recent studies (Brison et al., 2019; Ji et al., 2020; Macheret et al., 2020). Repli-seq revealed that there were generally two DNA peaks at the both sides of large CFSs such as FRA16D (*WWOX*) (Figure 2), indicating that the core region of CFSs failed to be replicated in late S phase under stressed conditions. In MiDASeq, the twin peaks of mitotic synthesized DNA would eventually converge, suggesting that stalled forks restart and progress inward to complete replication in accordance with BIR. In summary, stalled replication forks at CFSs are cleaved by MUS81-EME1 complex, leading to the formation of DSB. Thereafter, the two head-on forks carry out MiDAS to finish the replication of CFSs through BIR, which is dependent on POLD3 and RAD52 (Figure 3).

### 4.3 Transcription-Replication Interaction

The role of transcription in CFSs fragility may be traced back to the observations that CFSs were cell type specific. Murano *et al.* first showed the distribution and breakage frequency of CFSs substantially differ among lymphocytes, bone marrow cells and skin fibroblasts (Murano et al., 1989a; b). For example, the breakage frequency of FRA3B is 18.1% in lymphocytes, but decreases to 2.7% in fibroblasts. Similarly, Tallec *et al.* profiled CFSs expression in a wealth of cell types including epithelial cells, erythroid cells and mouse embryonic fibroblasts, and found that different epithelial cells share nearly 36% of their expressed CFSs, whereas that percentage for epithelial and erythroid cells is only 14% (Le Tallec et al., 2013). Considering that CFSs in varied cell types of the same species share identical genetic sequences, these similarities and dissimilarities in CFSs breakage could only be explained by epigenetic regulations. More recently, the cell type-specific transcription, especially that of CFSs-associated large genes, has been shown to predict the expression of a CFS in different cell lines, pointing to a mechanistic role of transcription in CFSs fragility.

Numerous studies have revealed that CFSs reside within chromosome regions hosting large genes over 300 kb long (Smith et al., 2006; McAvoy et al., 2007; Smith et al., 2007; Helmrich et al., 2011; Le Tallec et al., 2013; Okamoto et al., 2018; Pentzold et al., 2018). Typically, the extremely unstable FRA3B and FRA16D locate within the 1.5 Mb *FHIT* gene and 1.1 Mb *WWOX* gene, respectively (Ohta et al., 1996; Bednarek et al., 2000). It has been observed that CFSs in different species can overlap with the same orthologous large genes, indicating that the conservation of CFSs across species originates from evolutionarily conserved large genes. Despite the strong correlation, not all of the large genes are CFSs related. Several lines of evidence have shown that only actively transcribed large genes are correlated with chromosome fragility (Le Tallec et al., 2013; Okamoto et al., 2018; Pentzold et al., 2018). A pioneering work focusing on five CFSs-related genes reported that FRA3B and FRA16D are broken only in B-lymphoblasts, where the associated *FHIT* and *WWOX* genes are actively transcribed, but stay stable in myoblast where *FHIT* and *WWOX* genes are silent (Helmrich et al., 2011). It should, however, be noted that the vast majority of large transcribed genes do not exhibit fragility, as revealed by the RNA-seq data from HCT116 cells and avian cells (Le Tallec et al., 2013; Pentzold et al., 2018), which suggests that transcription can only cause CFSs fragility under restricted conditions. Wilson *et al.* used Bru-seq to show for the first time that only large transcripts, corresponding to the longest expressed isoform of large genes, are correlated with CFSs fragility (Wilson et al., 2015). This is further supported by two recent studies which conducted genome-wide mapping of CFSs through ChIP-seq of CFSs-binding protein FANCD2 (Okamoto et al., 2018; Pentzold et al., 2018). The genomic CFSs landscape provided more comprehensive and convincing evidence showing that most CFSs-associated genes are actively transcribed into long transcripts distributed across the gene body, even though the RNA level of some transcripts are relatively low. There appears to be a causal relationship between the large transcripts of large genes and CFSs fragility. In an attempt to experimentally address the impact of transcription on CFSs fragility, Blin *et al.* utilized a Tet-on inducible system to directly manipulate the transcription level of large genes and examined the changes in replication and breakage patterns of CFSs (Blin et al., 2019). They found that upregulation of the DMD transcription (a silent stable large gene) caused its fragility, replication fork slowing but its replication timing remaining unaffected. Interestingly, transcription can also have opposite effects on CFSs expression, as shown that overexpression of FRA4F-associated *CCSER1* or FRA3B-associated *FHIT* mitigated CFSs instability by advancing replication timing. These results indicate that the effects of transcription on replication depend on the context factors, e.g., the overall transcription level and the local epigenetic landscape.

Currently, two hypotheses on how transcription impacts CFSs fragility are actively being investigated, referred to “transcription-replication collision” and “replication origin modulation”. Large CFSs associated genes spanning over 800kb, such as *FHIT*, *WWOX,* and *IMMP2L*, require more than one cell cycle to complete their transcription that tends to initiate at the G2/M phase and persist into the next G1/S phase. It is thus easily inferred that certain subregions within these genes can experience concurrent transcription and replication especially under replicative stress, resulting in collision between the transcribing RNA polymerase II and the replication machinery, leading to replication stalling and the formation of R loops, an abnormal structure consisting of an RNA-DNA hybrid and a single-stranded DNA (Santos-Pereira and Aguilera, 2015; Crossley et al., 2019). R loops are thermodynamically stable and can persist for a long time, which may in turn exacerbate replication fork stalling. They are found to accumulate specifically at CFSs during mild replication stress and are predictive of high breakage frequency of CFSs (Okamoto et al., 2018; Wu et al., 2020). Besides, the mRNA expression levels of some CFSs associated genes, such as *WWOX*, are also positively correlated with CFSs instability. These findings strongly support the “transcription-replication collision” model. From this model, it is reasonable to assume that CFS breakage sites are located within transcribed genes, and that high transcription levels enhance the frequency of CFSs breakage by raising the chance of replication-transcription collision. However, molecular mapping of CFSs by Le Tallec *et al.* revealed that there were some CFSs whose breakage sites nested partly outside the transcribed genes (Le Tallec et al., 2013), and no correlation was found between the mRNA levels of large genes and their instability in HCT116 cells (Le Tallec et al., 2013). Moreover, Brison *et al.* found that transcription inhibition did not rescue CFSs fragility upon replication stress (Brison et al., 2019), providing the first direct evidence against the “transcription-replication collision” model. These findings suggest that transcription-replication encounter and the resultant R loop may not be the direct cause of CFSs instability.

Using nascent RNA sequencing, Wilson *et al.* found that CFSs were enriched in large transcription units, i.e., actively transcribed long isoform of large genes in human 090 fibroblast (Wilson et al., 2015). This trend has also been observed in U2OS cell line by analyzing the transcription profile of CFSs determined by MiDASeq (Ji et al., 2020). Importantly, it was shown that transcription can regulate the distribution and density of replication origins (Gros et al., 2015; Powell et al., 2015; Macheret and Halazonetis, 2018; Chen Y.-H. et al., 2019; Liu et al., 2021). These data lead to the second model named “replication origin modulation” in which large transcription units prevent dormant origin from firing upon replication stress by displacing pre-replication complex (pre-RC) within CFSs during G1 phase. In this scenario, CFSs can only be replicated by two flanking forks that need to travel a long distance to converge, as discussed earlier in the “double fork failure” model, making CFSs vulnerable to replication slowing or stalling.

### 4.4 Topological Tension in 3-Dimensional Genome

Although the combined effects of large transcription units, origin deficiency, and late replication timing seem to be able to explain the fragility of a large portion of CFSs, there are still chromosome regions that have all of these properties yet remain stable, prompting researchers to seek the nature of CFSs fragility in the context of three-dimensional (3D) genome configuration. Most recently, Sarni *et al.* analyzed the relationship between CFSs and topologically associated domains (TADs) in immortalized human foreskin fibroblasts (Sarni et al., 2020). Their results show that most of the late-replicated CFSs hosting large transcribed genes coincide with TAD boundaries. The replication timing of CFSs overlapping with TAD boundaries is much more delayed than that of non-overlapping ones. Furthermore, TAD boundaries are exactly located at the core region of CFSs experiencing the most delayed replication timing. However, simply overlapping with TAD boundaries per se is not enough to elicit instability, as stable genomic regions linked to TAD boundaries are prevalent across the genome. The association of CFSs with TAD boundary highlights the role of topological tension from 3D genome organization in chromosomal fragility and genomic stability.

## 5 Conclusion and Future Perspectives

CFSs manifest as the most dangerous form of genomic instability, chromosome breakage right before allocation of genetic materials into two daughter cells, which intimately correlate with some notorious genomic abnormalities such as CNV. Since the discovery of CFSs, underlying fragility mechanism has been at the forefront of CFSs research. To address this question, the first step would be identifying their genomic locations, which will allow for the acquisition of their genetic sequences and functional studies in the regions of interest. For decades, FISH has been the main approach to mapping and characterizing CFSs at the molecular level, laying the foundation for most of our current perceptions of CFSs. However, there is always a latent risk that those hypotheses or conclusions derived from FISH-based investigation on a very limited number of CFSs may be biased and only apply to a subset of CFSs. In comparison, genome-wide mapping of CFSs by high-throughput sequencing can provide accurate coordinates and genetic sequences for a large number of CFSs. As discussed earlier, although these high-throughput mapping technologies are based on current knowledge of CFSs, the comprehensive information from them has already refreshed our understanding of some aspects of CFSs with regard to their general sequence characteristics and replication dynamics. Hopefully, future studies can combine high-throughput mapping of CFSs with other complementary tools to gain deeper insight into CFSs and their biological relevance, which may lead us closer to the ultimate origin, if there is one, of CFSs fragility.
